# Chemical composition and antibacterial activities of seven *Eucalyptus* species essential oils leaves

**DOI:** 10.1186/0717-6287-48-7

**Published:** 2015-01-19

**Authors:** Khaled Sebei, Fawzi Sakouhi, Wahid Herchi, Mohamed Larbi Khouja, Sadok Boukhchina

**Affiliations:** Laboratoire de Biochimie des Lipides et Interactions avec les Macromolécules, Faculté des Sciences de Tunis, Université de Tunis–El-Manar, Elmanar, 2092 Tunis, Tunisia; Laboratoire d’Ecologie, INRGREF, B.P10, Ariana, 2080 Tunisia

**Keywords:** *Eucalyptus*, Essential oil, Antibacterial activity, α-pinene

## Abstract

**Background:**

In this paper, we have studied the essential oils chemical composition of the leaves of seven *Eucalyptus* species developed in Tunisia. *Eucalyptus* leaves were picked from trees growing in different arboretums in Tunisia. Choucha and Mrifeg arboretums located in Sedjnene, region of Bizerte (Choucha: *E. maideni, E. astrengens* et *E. cinerea;* Mrifeg : *E. leucoxylon*), Korbous arboretums located in the region of Nabeul, North East Tunisia with sub-humid bioclimate, (*E. lehmani*), Souiniet-Ain Drahem arboretum located in region of Jendouba (*E. sideroxylon, E. bicostata*). Essential oils were individually tested against a large panel of microorganisms including *Staphylococcus aureus* (ATCC 6539), *Escherichia coli* (ATCC 25922), *Enterococcus faecalis* (ATCC29212), *Listeria ivanovii* (RBL 30), *Bacillus cereus* (ATCC11778).

**Results:**

The yield of essential oils ranged from 1.2% to 3% (w/w) for the different *Eucalyptus* species. All essential oils contain α-pinene, 1,8-cineol and pinocarveol-trans for all *Eucalyptus* species studied. The 1,8-cineol was the major compound in all species (49.07 to 83.59%). Diameter of inhibition zone of essential oils of *Eucalyptus* species varied from 10 to 29 mm. The largest zone of inhibition was obtained for *Bacillus cereus* (*E. astrengens*) and the lowest for *Staphylococcus aureus (E. cinerea)*. The essential oils *from E. maideni, E. astrengens, E. cinerea* (arboretum of Bizerte), *E. bicostata* (arboretum of Aindraham) showed the highest antibacterial activity against *Listeria ivanovii and Bacillus cereus*.

**Conclusion:**

The major constituents of *Eucalyptus* leaves essential oils are 1,8-cineol (49.07 to 83.59%) and α-pinene (1.27 to 26.35%). The essential oils *from E. maideni, E. astrengens, E. cinerea*, *E. bicostata* showed the highest antibacterial activity against *Listeria ivanovii and Bacillus cereus*, they may have potential applications in food and pharmaceutical products.

## Background

*Eucalyptus*, a native genus from Australia, belongs to Myrtaceae family and comprises about 900 species and subspecies, it is one of the world’s most important and most widely planted genera [1–4]. It has been introduced worldwide, including in Tunisia and mainly cultivated for its timber, pulp and essential oils that present medicinal properties and therapeutic uses [5]. In recent decades, the essential oils and their components of plants have been of great interest as they have been the sources of natural products [6]. The value of *Eucalyptus* oil for medicinal purposes is based largely on the content of a particular oil constituent: 1,8-cineole (cineole or eucalyptol) [7]. Hot water extracts of dried leaves of *Eucalyptus citriodora* are traditionally used as analgesic, anti-inflammatory and antipyretic remedies for the symptoms of respiratory infections, such as cold, flu, and sinus congestion ([8], *Eucalyptus camaldulensis* and *Eucalyptus urophylla* are also known to contain bioactive products that showed antibacterial [9], antifungal [10], analgesic and anti-inflammatory effects [8], antioxidative and antiradical [11] activities. Various studies showed that differences in the yield and composition of the essential oils were influenced by the time of harvest, moreover, for *Eucalyptus* genus, the amount and composition of leaf oil may vary seasonally and diurnally for some plants, depending on environmental conditions [12].

The objectives of the present study are to determine the chemical composition of the essential oils of seven common Tunisian *Eucalyptus* species, namely *E. lehmani*; *E. leucoxylon*; *E. astrengens*; *E. cinerea*; *E. maideni*; *E. sideroxylon*; *E. bicostata*. The study also aims at investigating the antibacterial properties against some of the common pathogens bacteria. In addition, the study determines the influence of growth conditions on the chemical composition and antibacterial properties of the essential oils of *Eucalyptus* species.

## Results and discussion

### *Eucalyptus*essential oil yields

The yield of essential oils ranged from 1.2% to 3% (w/w) for the different *Eucalyptus* species (Table 1). The highest yield was obtained from *E. cinerea* and *E. sideroxylon* (3%), followed by *E. lehmani* (2.8%), while *E. astrengens* gave the lowest yield at 1.2%. According to these results, we confirmed that there is no relationship between region and *Eucalyptus* essential oil yield. In fact, species from the same region show different yields (*E. leucoxylon*; *E. astrengens*; *E. cinérea*) while species from different regions have almost the same yield.

**Table 1:** ***Eucalyptus*** **essential oil yield at the different regions of Tunisia**

	E.m	E.a	E.c	E.le	E.l	E.s	E.b
**Yield of essential oil (%)**	1.5	1.2	3	1.6	2.8	3	2

E.m : *E. maideni*; E.a : *E. astrengens*; E.c : *E. cinerea*; E.le : *E. leucoxylon*; E.l : *E. lehmani*; E.s : *E. sideroxylon*; E.b : *E. bicostata*.

Some parameters can influence yields such as leaves age [13], the harvest date [14], geographical origine [15], distillation method [16, 17]^.^ Ben Jemàa et *al*., [12] reported that essential oil yields varied according to *Eucalyptus* species and seasons and for all species studied, (*E. camaldulensis, E. astringens, E. leucoxylon, E. lehmannii and E. rudis*) high yields were obtained from leaves collected at the summer season though *E. astringens* gave rather constant yields during winter (1.23% for spring and 1.1% for winter).

### Composition of the essential oils

The chemical composition of the essential oils extracted from *Eucalyptus* species (*E. maideni; E. astrengens; E. cinerea; E. leucoxylon; E. lehmani; E. sideroxylon; E. bicostata*) is presented in Table 2. All essential oils contain α-pinene, 1,8-cineol and pinocarveol-trans for all *Eucalyptus* species studied. The 1,8-cineol was the major compound in all species. The essential oil composition of the *Eucalyptus* species from the region of Bizerte showed that all of them contained 1,8-cineole, the highest content was obtained from *E. maideni* (83.59%) followed by *E. cinerea* and *E. lehmani* (respectively 79.18% and 49.07%), while *E. astrengens* gave the lowest rate (60.01%). Although these species are from the same region, they show differences in the levels of some essential oil compounds. This may be due to genetic effects. Essential oils extracted from species from Aindraham arboretum (*E. sideroxylon and E. bicostata*) have the same 1,8 cineole level and the species from Korbous arboretum (*E. lehmani*) have the lowest rate of 1,8-cineole (49.07%) and the highest level of α-pinene (26.35%). We could identify other compounds of relatively high rates such as globulol and pinacarvone. Ben jemâa et *al*., [12] reported that GC and GC-MS analyses showed that chemical composition varied with Tunisian *Eucalyptus* species and seasons. The five essential oils contained 1,8-cineole, α-pinene, and α-terpineol as major common compounds. The essential oils of twenty *Eucalyptus* species harvested from North West and North of Tunisia were studied and the authors identified, by GC and GC/MS, eighteen major compounds and the main ones were 1,8-cineol followed by α-pinene, p-cymene, borneol, cryptone, spathulenol, viridiflorol and limonene. The authors showed that main components were oxygenated monoterpenes, among them 1,8-cineol which was the major one in leaf essential oils of ten species, followed by trans-pinocarveol and α-terpineol. The oxygenated sesquiterpenes were the second major class represented essentially by borneol, spathulenol, viridiflorol and globulol; the third major class was the monoterpene hydrocarbons constituted by a high level of α-pinene, p-cymene and limonene [16].

**Table 2:** **Chemical composition of essential oil from** ***Eucalyptus*** **leaves**

***Compounds (%)***	***E.m***	***E.a***	***E.c***	***E.le***	***E.l***	***E.s***	***E.b***
α- pinene	1.27	6.96	4.08	5.85	26.35	5.81	2.16
1,8-cineol	83.59	60.01	79.18	77.76	49.07	80.75	81.29
Terpineol alpha	-	-	2.20	-	3.51	2.45	-
Cymene P	-	2.31	-	-	2.42	-	-
Terpinene gamma	-	-	-	2	1,58	-	-
Trans-Pinocarveol	3.40	8.92	2.07	3.23	1.59	1	4.49
Terpinyl acetate alpha	-	-	5.43		5.64	2.30	-
globulol	3.61	3.74	-	1.42	1.01	-	1.81
Limonene	-	-	-	1.33	-	3.32	-
Pinacarvone	1.28	4.70	-	1.15	-	-	3.93
Guaiene	-	1.33	-	-	-	-	-
Spathulenol	-	1.15	-	-	-	-	-
Mentha -1(7),8-Dien-2-ol trans P	-	-	1.09	-	-	-	1.03
**Others**	6.85	10.88	5.95	7.26	8.8	4.62	5.29

E.m : *E. maideni*; E.a : *E. astrengens*; E.c : *E. cinérea*; E.le : *E. leucoxylon*; E.l : *E. lehmani*; E.s : *E. sideroxylon*; E.b : *E. bicostata*.

In Taiwanian *Eucalyptus* species, a total of 20 compounds amounting to 97.58% in the *E. camaldulensis* leaf essential oil were identified. Among these 81.41% were monoterpene hydrocarbons, 12.55% were oxygenated monoterpenes, and it also contained 0.50% sesquiterpene hydrocarbons and 3.12% oxygenated sesquiterpenes. The major constituents in the *E. camaldulensis* leaf essential oil were α-pinene (22.52%), p-cymene (21.69%), aphellandrene (20.08%), 1,8-cineole (9.48%), c-terpinene (9.36%), and limonene (4.56%) [18]. For *Eucalyptus* species, many factors may influence monoterpene emissions, especially seasonal and diurnal emission activity cycles [19].

### Antibacterial activity

According to the zone diameter inhibition (zdi) values expressed in mm, results were ranked as follows: not sensitive (–) for zone diameters equal to 8 mm or below; sensitive (+) for zone diameters between 8 and 14 mm, very sensitive (++) for zone diameters between 14 and 20 mm and extremely sensitive (+++) for zone diameters equal or larger than 20 mm [17, 20, 21]. The results revealed that the essential oils showed antibacterial activity with varying magnitude and depending on the size of inoculums. Diameter of inhibition zone of essential oils of eucalyptus species varied from 10 to 29 mm (Table 3). The largest zone of inhibition was obtained for *Bacillus cereus* (*E. astrengens*) and the lowest for *Staphylococcus aureus (E. cinerea)*. The essential oils from *E. maideni, E. astrengens, E. cinerea* (arboretum of Bizerte), *E. bicostata* (arboretum of Aindraham) showed the highest antibacterial activity against *Listeria ivanovii and Bacillus cereus*.

**Table 3:** **Antibacterial activity of essential oil of** ***Eucalyptus*** **species**

Strains	E.m	E.a	E.c	E.le	E.l	E.s	E.b
***Listeria ivanovii*** **(RBL30)**	25 mm +++	26 mm +++	24 mm +++	12 mm +	11 mm +	20 mm ++	28 mm +++
***Escherichia coli*** **(ATCC25922)**	15 mm ++	15 mm ++	16 mm ++	18 mm ++	20 mm ++	14 mm +	--
***Staphylococcu*** **s** ***aureus*** **(ATCC6533)**	--	13 mm +	10 mm +	11 mm +	16 mm ++	12 mm +	15 mm ++
***Enterococcus faecalis*** **(ATCC29212)**	11 mm +	12 mm +	12 mm +	12 mm +	12 mm +	11 mm +	10 mm +
***Bacillus cereus*** **(ATCC11778)**	25 mm +++	29 mm +++	21 mm +++	16 mm ++	17 mm ++	15 mm ++	27 mm +++

Not sensitive (–) for zone diameters equal to 8 mm or below; Sensitive (+) for zone diameters between 8 and 14 mm, Very sensitive (++) for zone diameters between 14 and 20 mm, Extremely sensitive (+++) for zone diameters equal or larger than 20 mm.

E.m : *E. maideni*; E.a : *E. astrengens*; E.c : *E. cinérea*; E.le : *E. leucoxylon*; E.l : *E. lehmani*; E.s : *E. sideroxylon*; E.b : *E. bicostata*.

## Conclusion

The *Eucalyptus* species investigated in the present study show a large variation in their chemical composition. The major constituents of *Eucalyptus* leaves essential oils are 1,8-cineol (49.07 to 83.59%) and α-pinene (1.27 to 26.35%).

The essential oils *from E. maideni, E. astrengens, E. cinerea*, *E. bicostata* showed the highest antibacterial activity against *Listeria ivanovii and Bacillus cereus*, they may have potential applications in food and pharmaceutical products.

## Methods

### Collection of plant material

*Eucalyptus* leaves were picked from trees growing in different arboretums in Tunisia: Choucha and Mrifeg arboretums located in Sedjnene, region of Bizerte (Choucha: *E. maideni, E. astrengens* and *E. cinerea;* Mrifeg: *E. leucoxylon*), Korbous arboretum located in the region of Nabeul, (*E. lehmani*), Souiniet-Ain Drahem arboretum located in the region of Jendouba (*E. sideroxylon, E. bicostata*). The leaves were stored at a dry place for fifteen days. Specimens were identified at the Regional Station of the National Institute of Research in Farming Studies, Waters and Forests (INRGREF).

### Isolation of the essential oils

One hundred grams of dried leaves were submitted to water distillation (500 mL of water) for 3 hours, using a Clevenger-type apparatus. The obtained essential oils were dried over anhydrous sodium sulphate and after filtration, stored at 4 to 7°C until use. The extraction yield was calculated using the following formula: yield = (VEO × 100)/D.M (D.M: dry material; VEO: volume of essential oil).

### Gas chromatography analysis/mass spectrometry analysis

The GC analyses were accomplished with a HP-5890 Series II instrument equipped with HP-WAX and HP-5 capillary columns (both 30 m × 0.25 mm, 0.25 mm film thickness), working with the following temperature program: 60°C for 10 min, ramp of 5°C/min up to 220°C; injector and detector temperatures 250°C; carrier gas nitrogen (2 mL/min); detector dual FID; split ratio 1:30; injection of 0.5 mL). The identification of the components was performed, for both columns, by comparison of their retention times with those of pure authentic samples and by means of their linear retention indices (l.r.i.) relative to a series of n-hydrocarbons. The relative proportions of the essential oil constituents were percentages obtained by FID peak-area normalization, without using response factors.

For GC/MS detection, an electron ionization system, with ionization energy of 70 eV, a scan time of 1.5 s and mass range 40–300 amu, was used. Helium was the carrier gas at a flow rate of 1.2 mL/min. Injector and transfer line temperatures were set at 250 and 280°C, respectively. Oven program temperature was the same with GC analysis. Diluted samples (1/100 in hexane, v/v) of 1.0 μl were injected manually and in the splitless mode. The identification of the compounds was based on mass spectra (compared with Wiley 275.L, 6th edition mass spectral library) or with authentic compounds and confirmed by comparison of their retention indices either with those of authentic compounds or with data published in the literature as described by Adams [22]. Further confirmation was done from Kovats Retention Index data generated from a series of n-alkanes retention indices (relative to C9-C28 on the BP-1).

### Antibacterial activity detection

Essential oils were individually tested against a panel of microorganisms including *Staphylococcus aureus* (ATCC 6539), *Escherichia coli* (ATCC 25922), *Enterococcus faecalis* (ATCC29212), *Listeria ivanovii* (RBL 30), *Bacillus cereus* (ATCC11778). All strains were obtained from Institut Pasteur de Tunis. The Bacteriological agar was from Biokar Diagnostics (Beauvais, France). Nutrient broth (NB) was from Difco (Becton Dickinson, Le Pont de Claix, France). All the other media, used in this study, were manufactured by Biorad (Marnes-La Coquette, France) and Merck. Antibacterial activity is revealed by growth inhibition in the strains to test. This activity is observed in solid medium. In the present work, we used well diffusion method described by Perez et *al*., [23].

### Statistical analysis

The data (three replicates) were statistically evaluated using the JMP SAS version 12.6 software (Statistical Analysis System). (SAS, Institute INC, Box 8000, Cary, North Carolina 27511, USA).
